# Supplementary material for: Plasmodium falciparum CLAG Paralogs All Traffic to the Host Membrane but Knockouts Have Distinct Phenotypes
Source: Microorganisms. 2024 Jun 8;12(6):1172. doi: 10.3390/microorganisms12061172 (PMC11205492; doi:10.3390/microorganisms12061172)
Supplement: Supplementary file 1 [file microorganisms-12-01172-s001.zip › CLAG KOs Supplementary Figures revised.pdf]

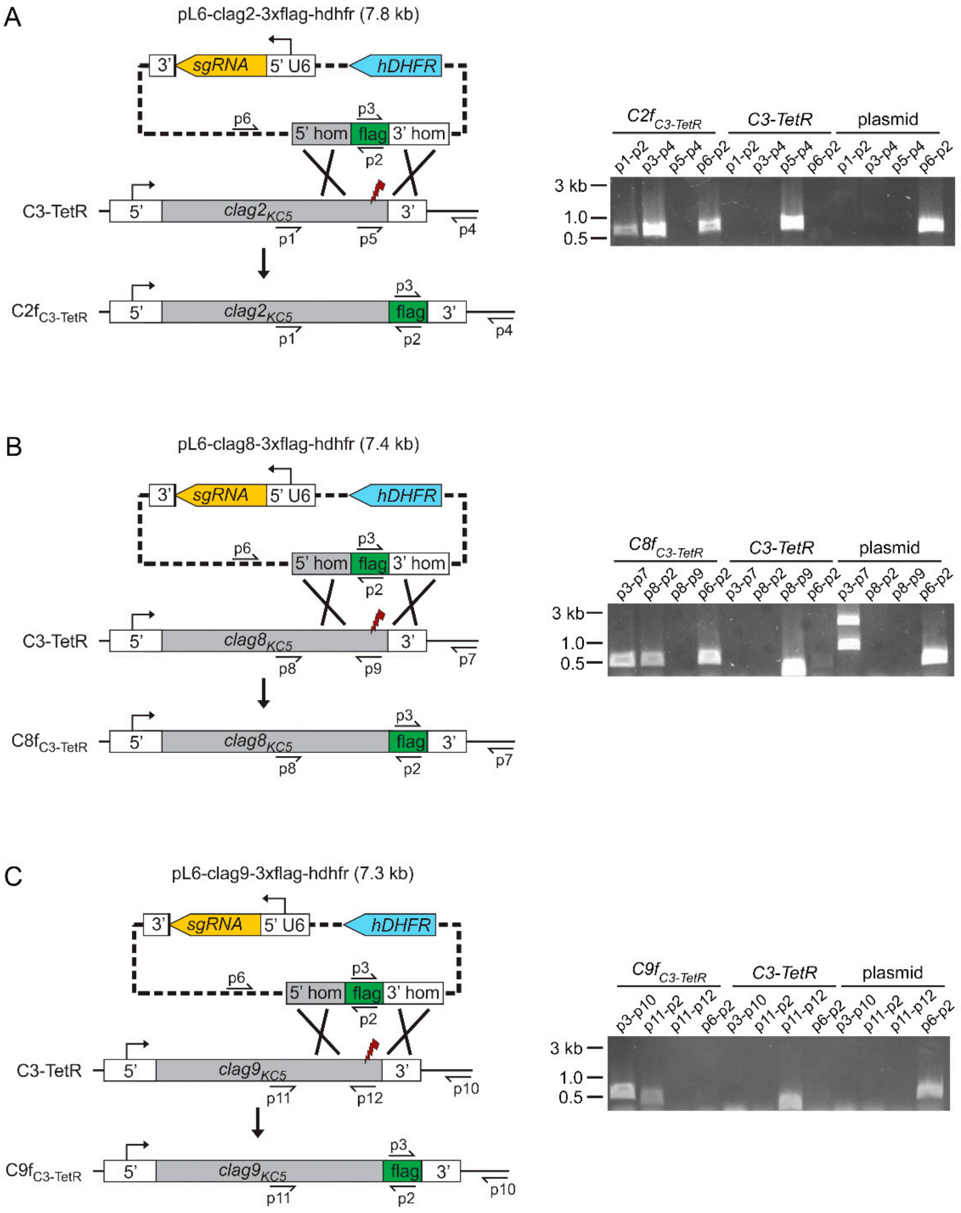

Figure S1. Strategy and production of double-tagged clones. **(A-C)** CRISPR-Cas9 transfection to produce *C2f*$_{C3\text{-}TetR}$, *C8f*$_{C3\text{-}TetR}$, and *C9f*$_{C3\text{-}TetR}$, respectively. In each panel, the schematic shows the transfection plasmid, homologous recombination at the genomic cleavage site, the resulting modification, and primer binding sites. Primers are listed in Table S1. Ethidium-stained gel at right shows PCR confirming integration (first two lanes, absence of residual wildtype sequence (third lane) and presence of the transfection episome (fourth lane). Control PCRs using the parental *C3-TetR* line and the transfection plasmid are also shown. Expected amplicon sizes (in bp) for panel A: p1-p2, 586; p3-p4, 582; p5-p4, 704; p6-p2, 603. For panel B: p3-p7, 483; p8-p2, 505; p8-p9, 357; p6-p2, 554. For panel C: p3-p10, 552; p11-p2, 440; p11-p12, 346; p6-p2, 500. The two bands seen with plasmid in the p3-p7 lane reflect spurious priming in the absence of cognate template. Cropped bands at the bottom of some lanes reflect primer dimers.

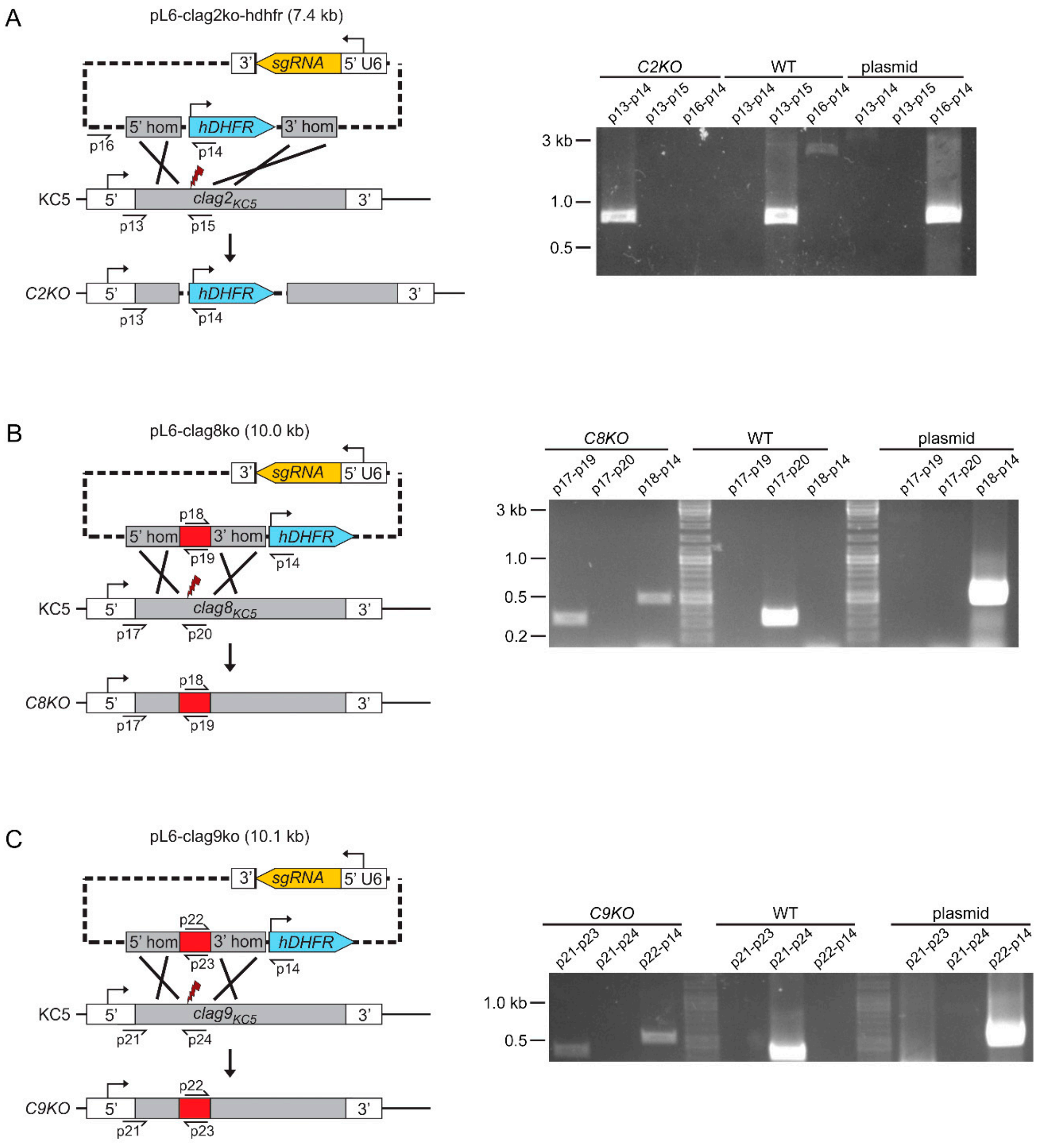

Figure S2. CRISPR knockout of CLAG paralogs. **(A-C)** Transfection to produce *C2KO, C8KO* and *C9KO* lines, respectively. Schematics show CRISPR-Cas9 mediated knockout of indicated *clag* paralogs and positions of primers (Table S1). Ethidium-stained gel in each panel shows PCR confirming integration to produce knockout (first lane), absence of residual wildtype sequence (second lane) and retention of the transfection episome (third lane). Control PCRs using the parental KC5 wild-type (WT) and the transfection plasmid are also shown. Expected amplicon sizes (in bp) for panel A: p13-p14, 750; p13-p15, 752; p16-p14, 750. For panel B: p17-p19, 301; p17-p20, 319; p18-p14, 483. For panel C: p21-p23, 392; p21-p24, 390; p22-p14, 530. Cropped bands at the bottom of some lanes reflect primer dimers.
