# Supplementary material for: Plasmodium falciparum CLAG Paralogs All Traffic to the Host Membrane but Knockouts Have Distinct Phenotypes
Source: Microorganisms. 2024 Jun 8;12(6):1172. doi: 10.3390/microorganisms12061172 (PMC11205492; doi:10.3390/microorganisms12061172)
Supplement: Supplementary file 1 [file microorganisms-12-01172-s001.zip › Uncropped blots for Gupta et al.pdf]

<u>Uncropped blots for Gupta *et al.*</u>

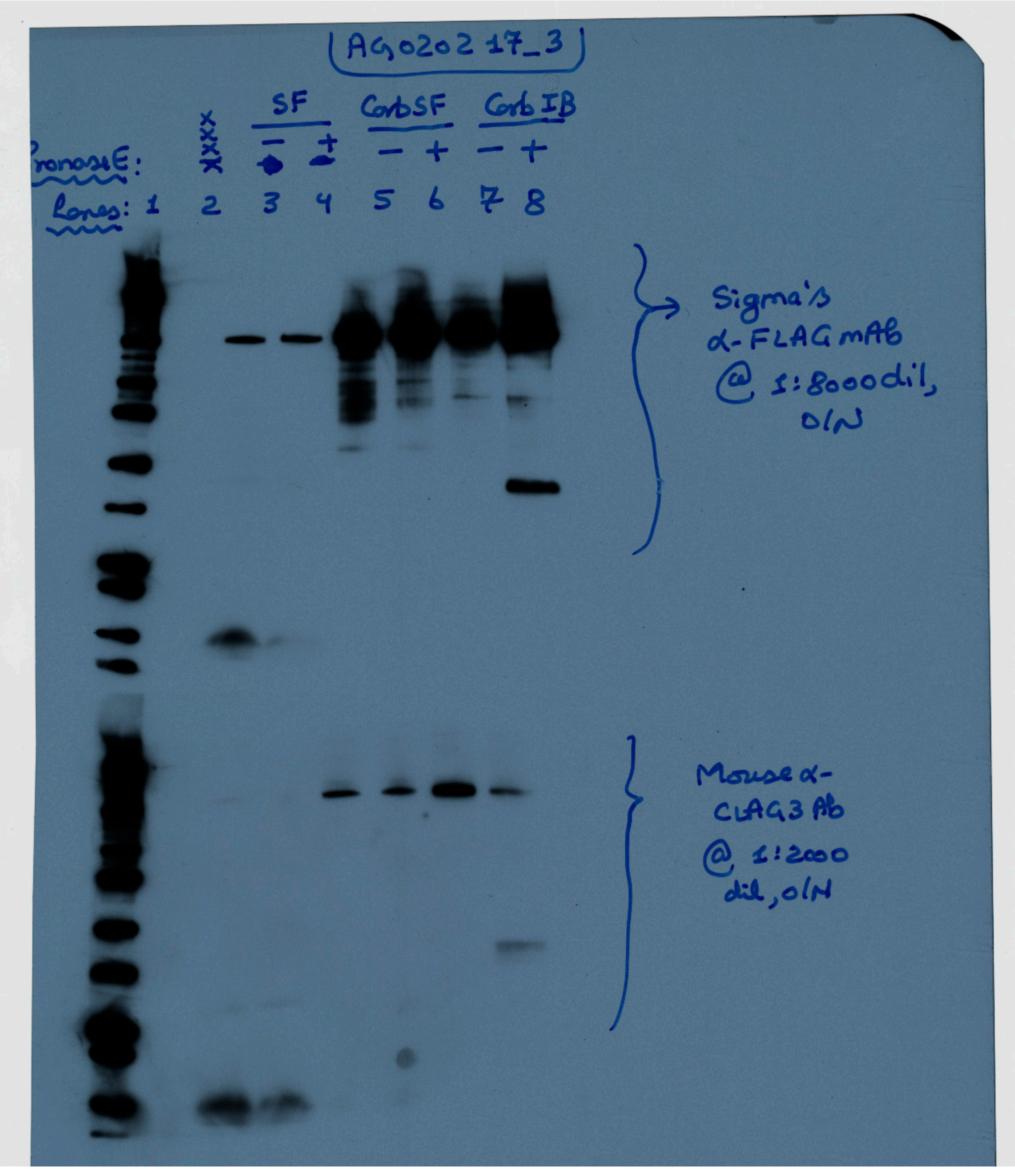

Figure 2A CLAG3 loading control for CLAG2 blot

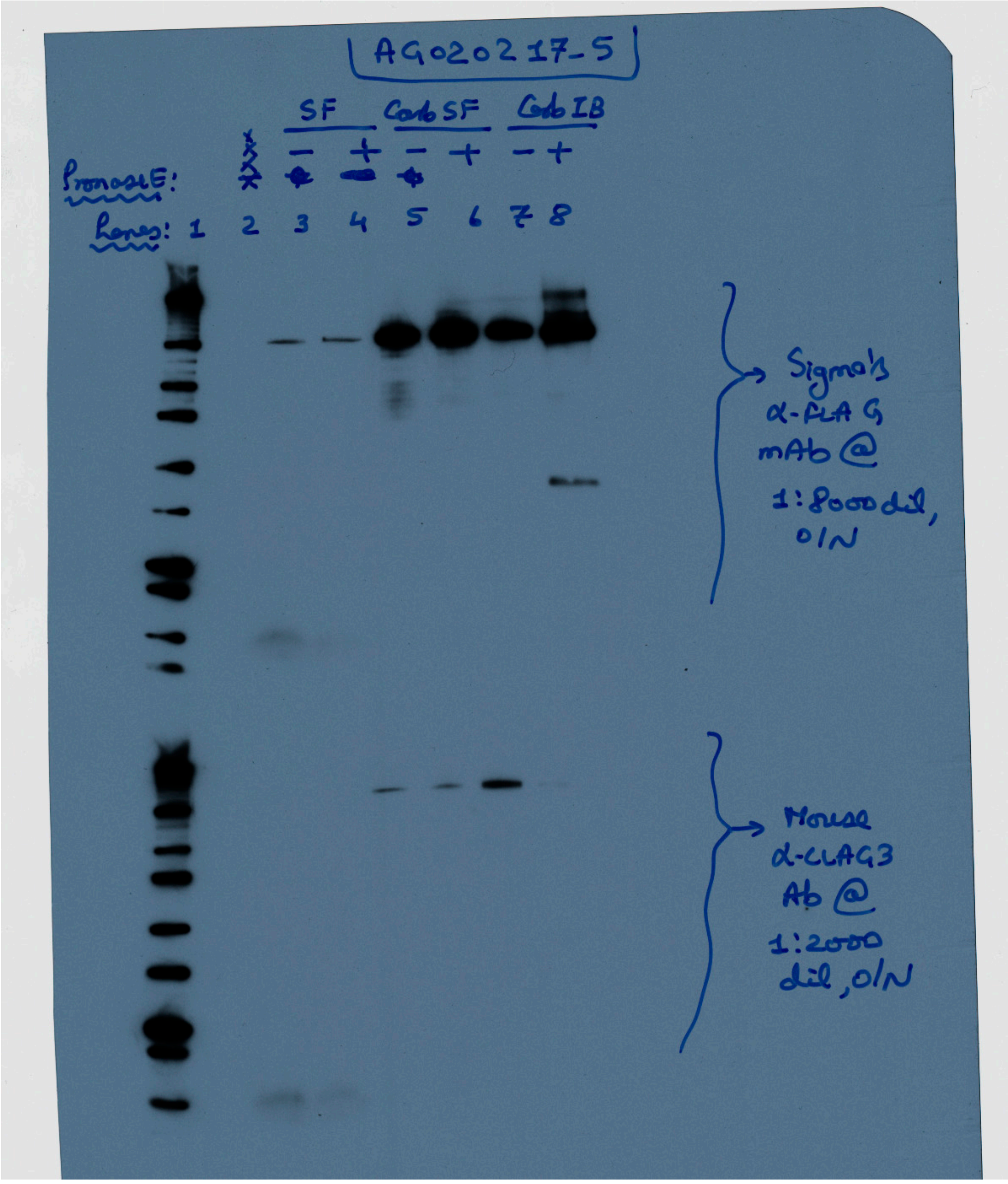

Figure 2A CLAG2

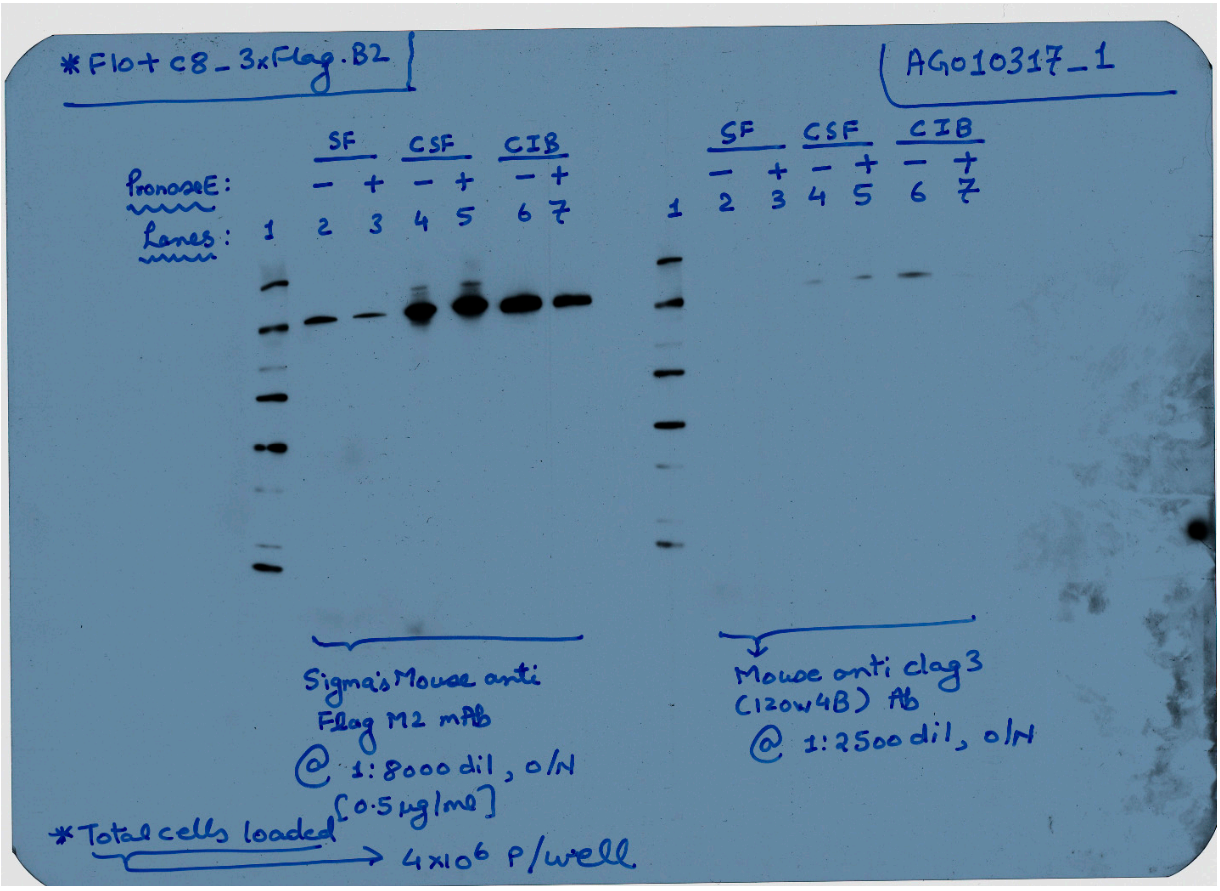

Figure 2A CLAG8

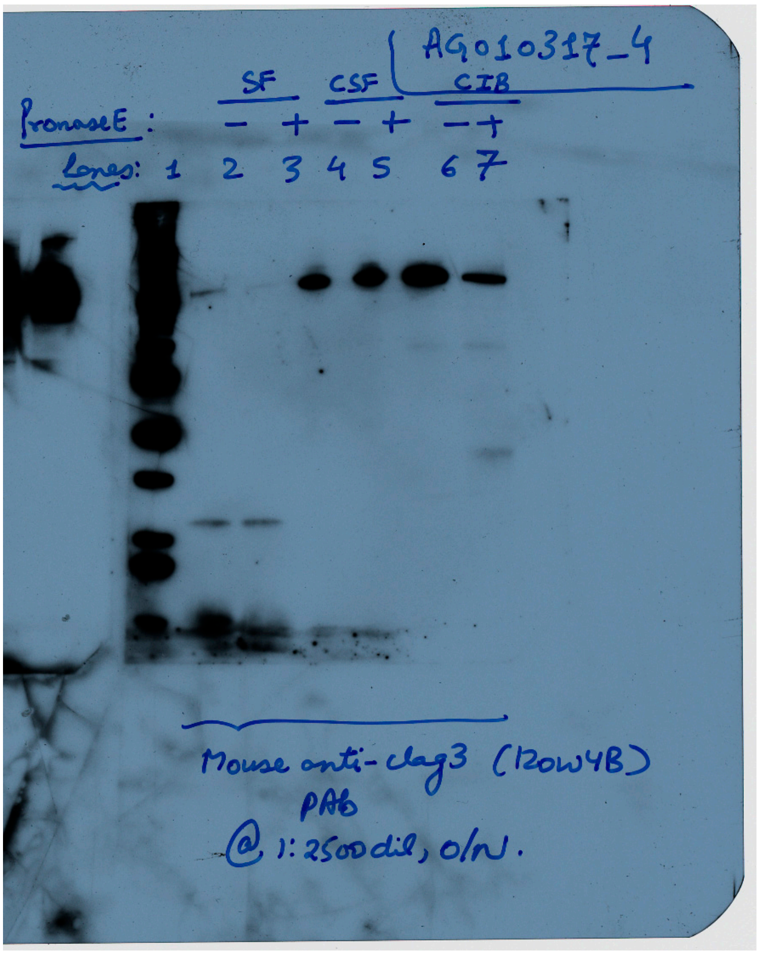

Figure 2A CLAG3 loading control for CLAG8 blot

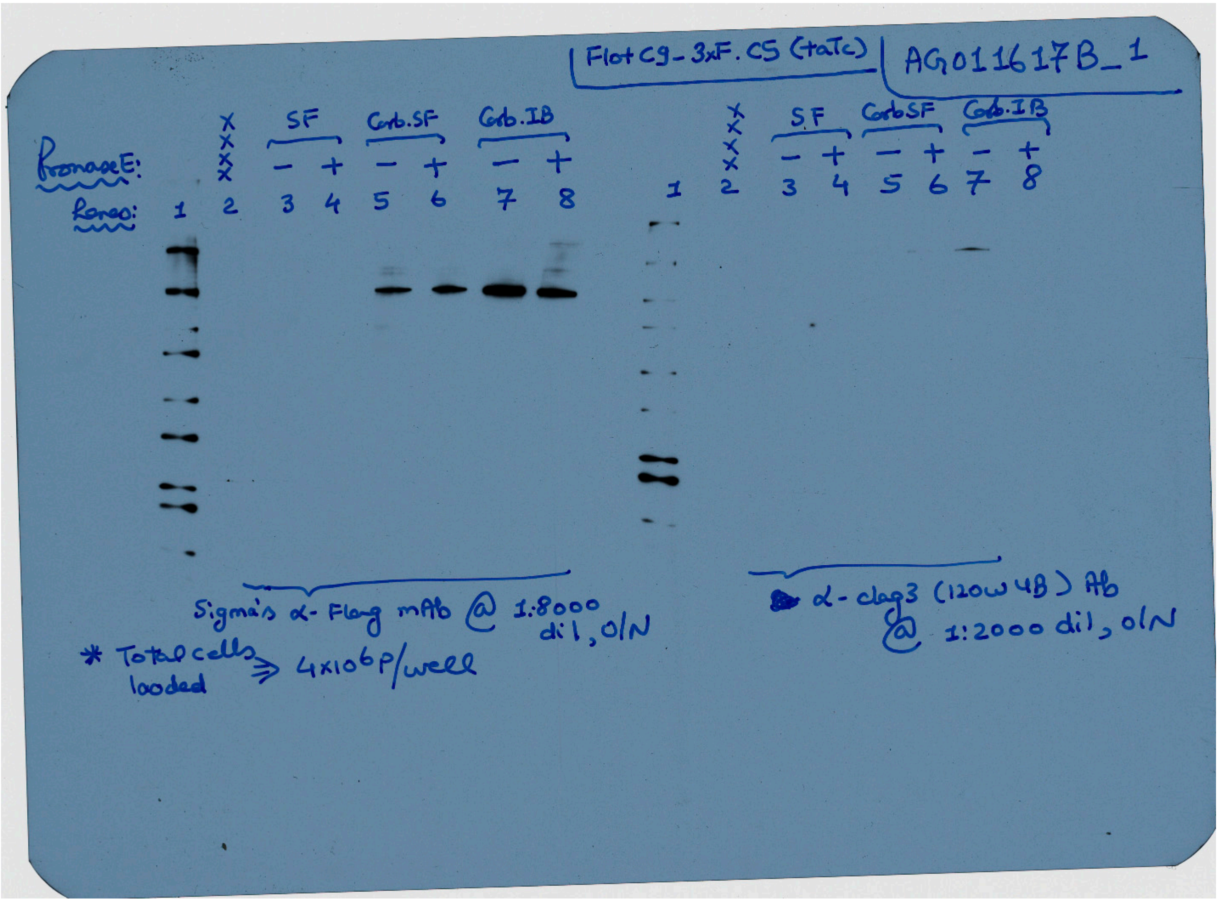

Figure 2A CLAG9

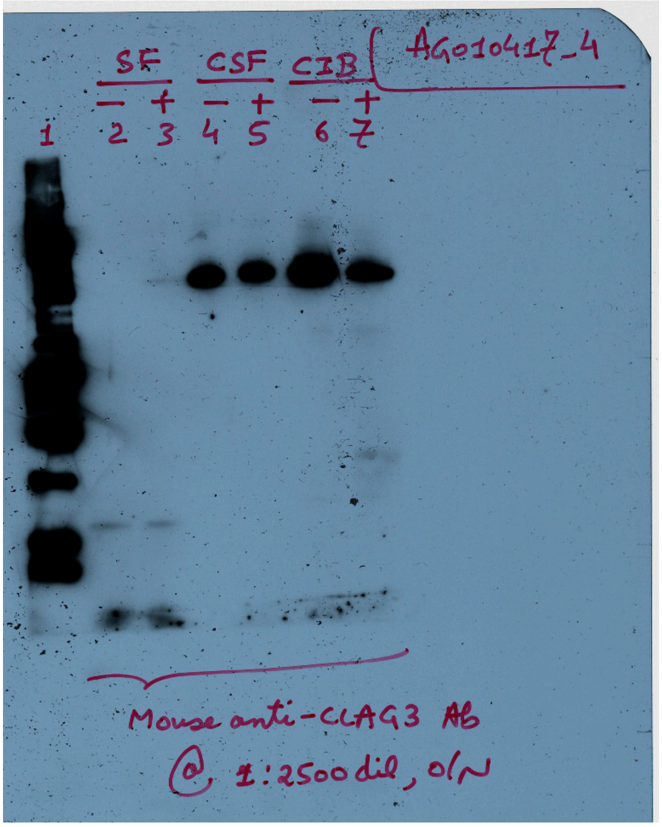

Figure 2A CLAG3 control for CLAG9 blot

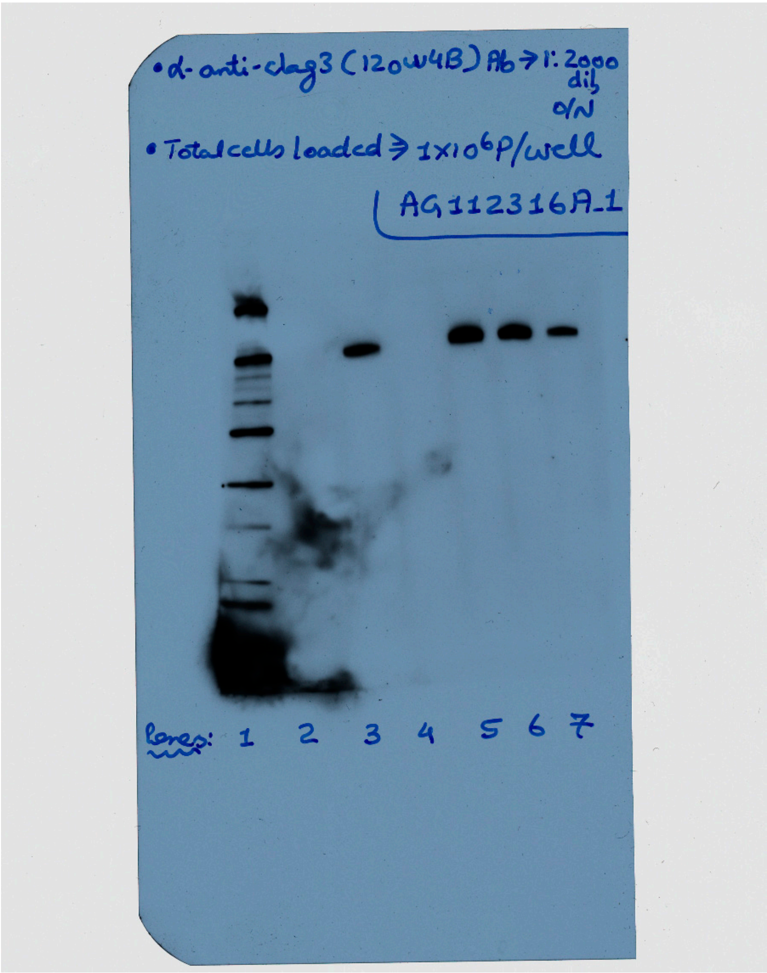

Figure 3B CLAG3

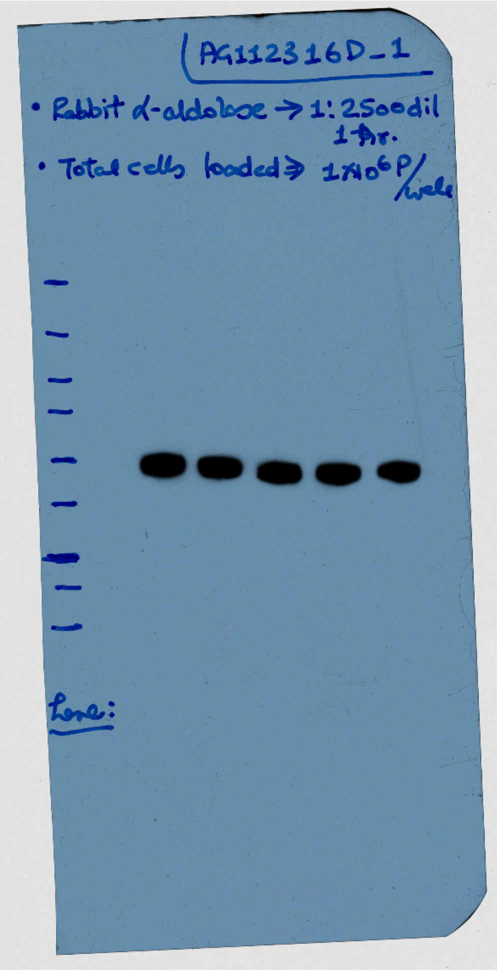

Figure 3B aldolase

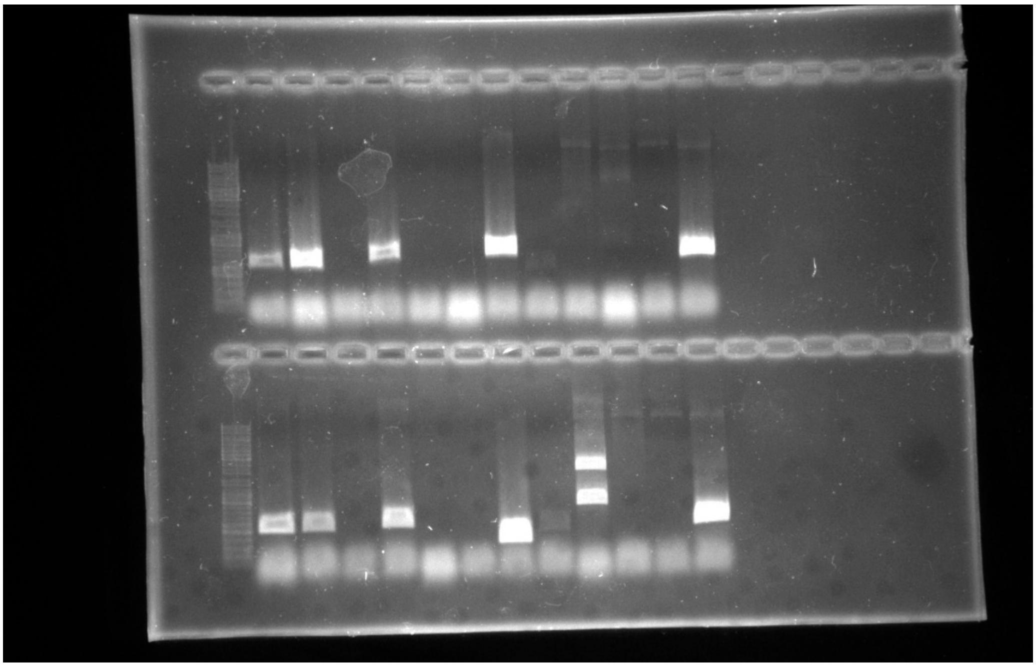

Figure S1 *clag2* and *clag8* PCR checks

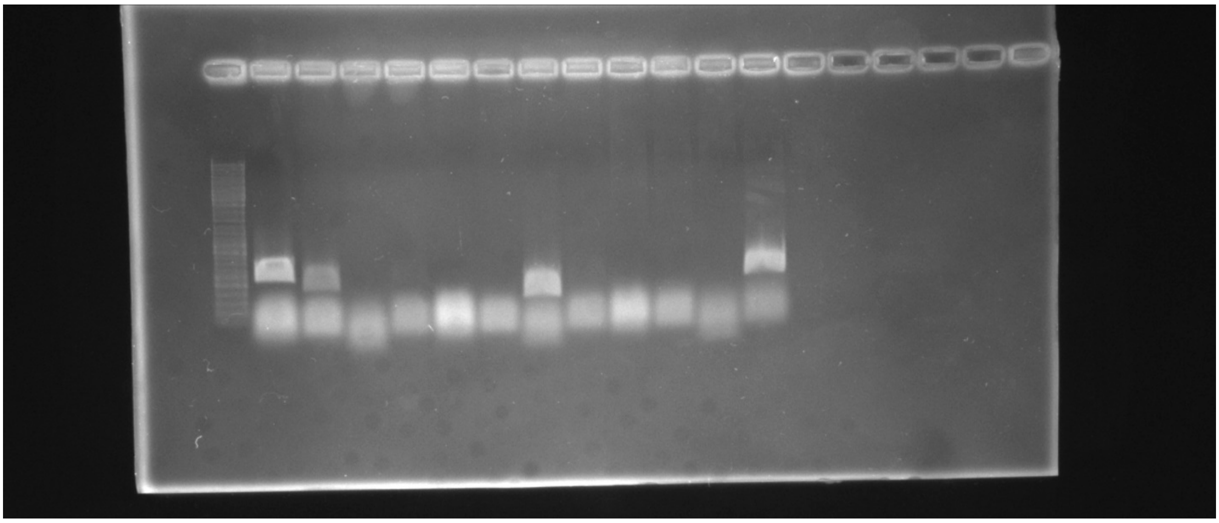
Figure S1 *clag9* PCR check

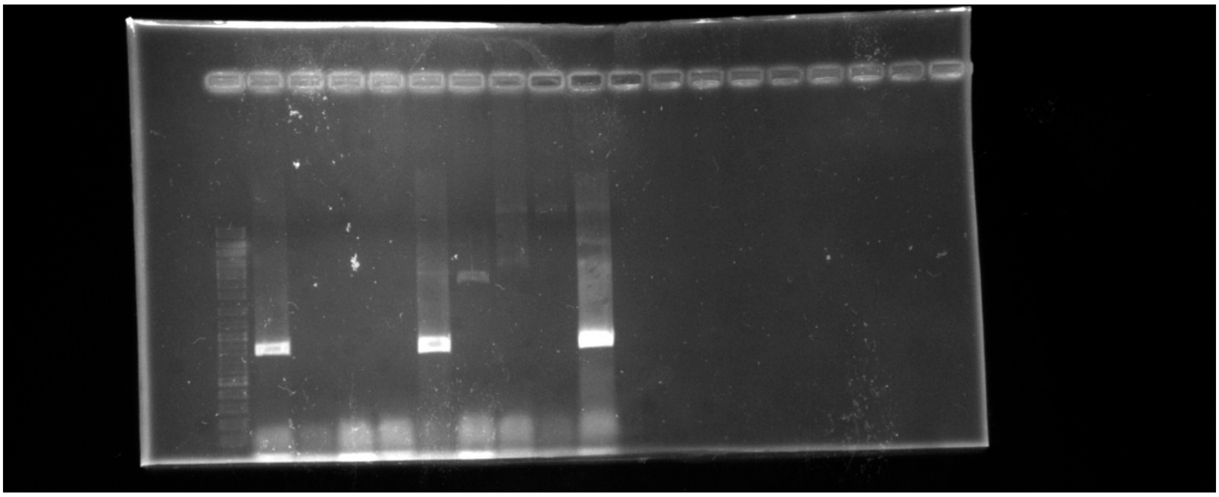
Figure S2 *clag2*

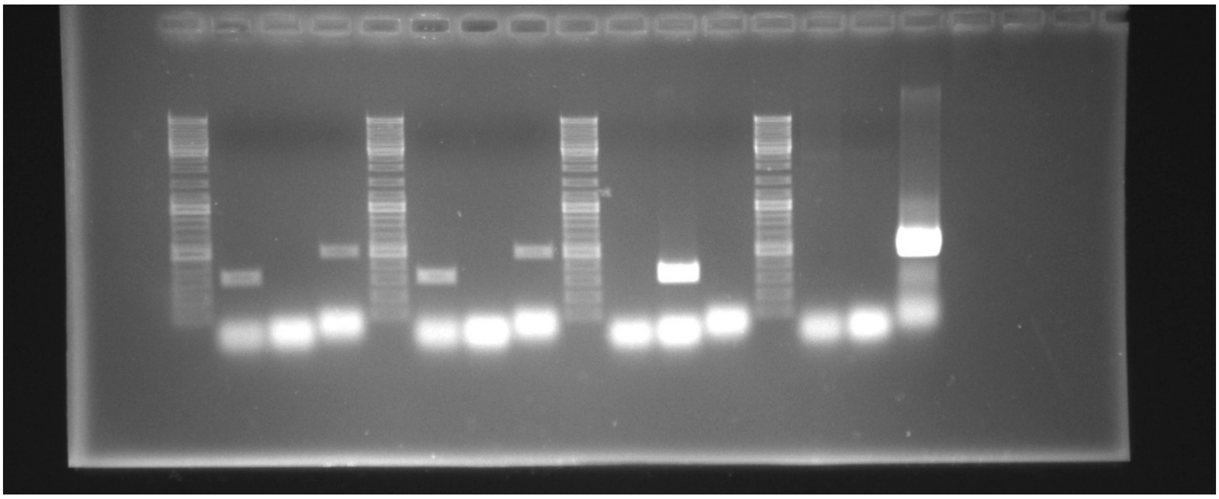
Figure S2 *clag8*

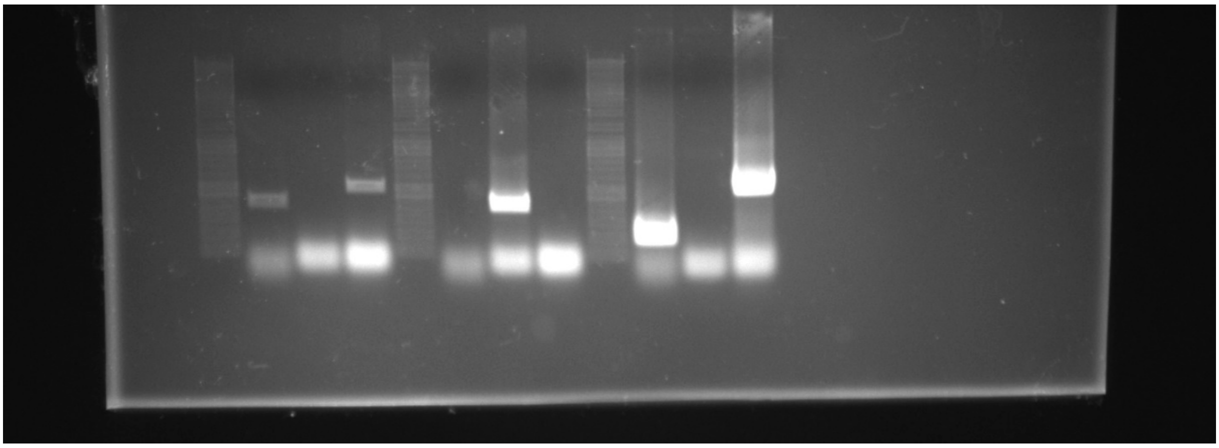

Figure S2 *clag9*
